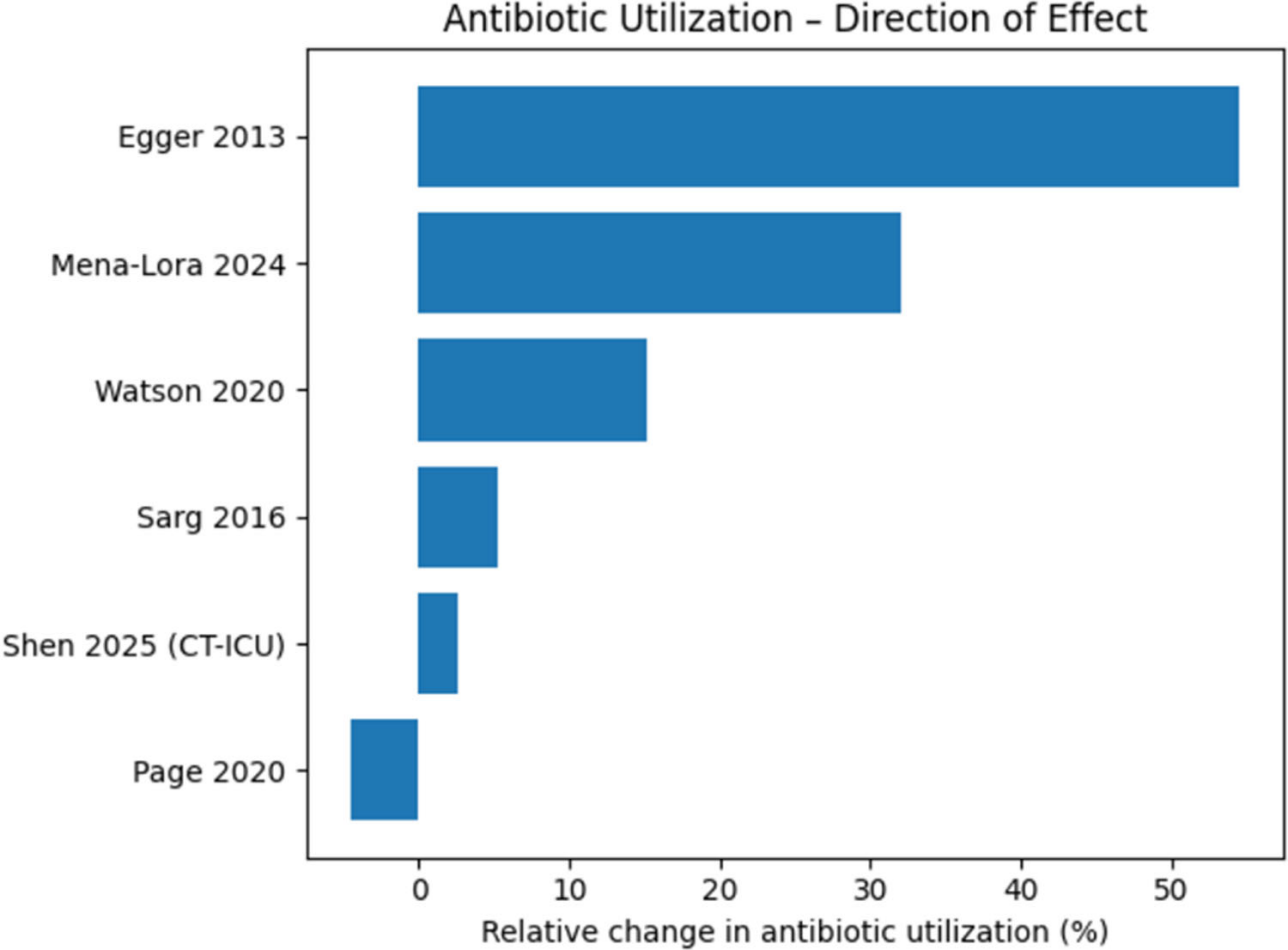# 148 Post-Vaccination Mumps. An Infrequent Adverse Event after Wide MMR Vaccination During an Outbreak of Measles in Mexico

**DOI:** 10.1017/ash.2026.10552

**Published:** 2026-06-23

**Authors:** Alexia El Khoury, Joy Abou Farah, Luke Kabbara, Laura Dee Presutti, Alina Abbas, Tina Lewis, Jay Krishnan, Elie Saade, University Hospitals Cleveland Medical Center

**Affiliations:** 1 Case Western Reserve University; 2 Case Western Reserve University/ UH Hospitals; 3 University Hospitals Health System; 4 University Hospitals, Case Western Reserve University

## Abstract

**Background:** Inappropriate urine culture ordering in hospitalized patients with indwelling urinary catheters contributes to unnecessary antibiotic exposure and increased risk of catheter-associated urinary tract infections (CAUTI). Urine Culture Stewardship Interventions have emerged as strategies to reduce unnecessary urine culture testing and downstream antibiotic use; however, the magnitude of their impact across clinical settings remains incompletely characterized. **Methods:** We conducted a scoping review following PRISMA-ScR guidelines to identify studies evaluating urine culture stewardship interventions in hospitalized adults (≥18 years) with indwelling urinary catheters. A systematic literature search was performed in PubMed, Cochrane Library, Web of Science, and Scopus from inception through June 2025, using predefined terms related to indwelling urinary catheters, urinary tract infections and CAUTI, urine culture and diagnostic stewardship, antibiotic use, and catheter management strategies. Interventions included reflex urine culture protocols, catheter replacement prior to specimen collection, educational initiatives, and multicomponent stewardship bundles. Outcomes of interest were urine culture utilization, CAUTI rates, and antibiotic utilization. Data were extracted independently by two reviewers using Covidence. Results were synthesized descriptively, and ranges of relative change were calculated by the review authors when pre- and post-intervention rates were explicitly reported. **Results:** **Conclusion:** Urine culture stewardship interventions in hospitalized adults with urinary catheters are consistently associated with meaningful reductions in urine culture utilization and CAUTI rates, with variable but generally favorable effects on antibiotic use. Education-based and reflex urine culture strategies, particularly when embedded within multicomponent stewardship bundles, appear most frequently studied and effective. These findings highlight the potential of diagnostic stewardship as a key component of infection prevention and antimicrobial stewardship efforts, while underscoring the need for standardized outcome reporting and to better quantify downstream clinical impact.

**Figure:**
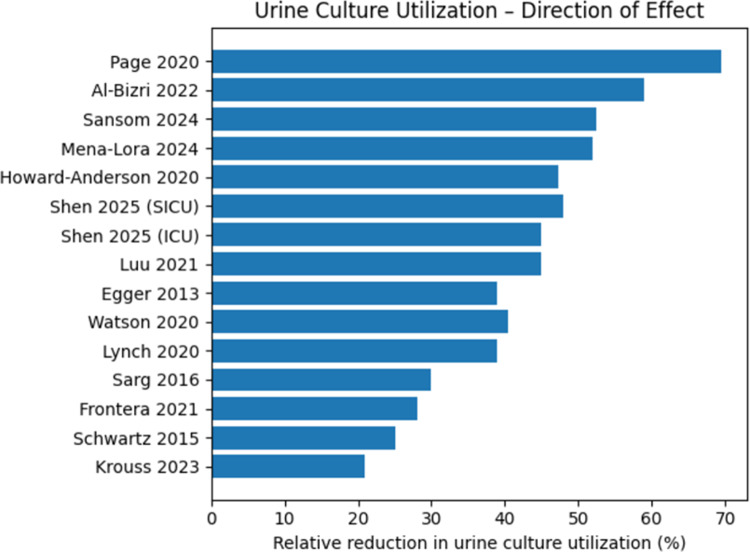

**Figure:**
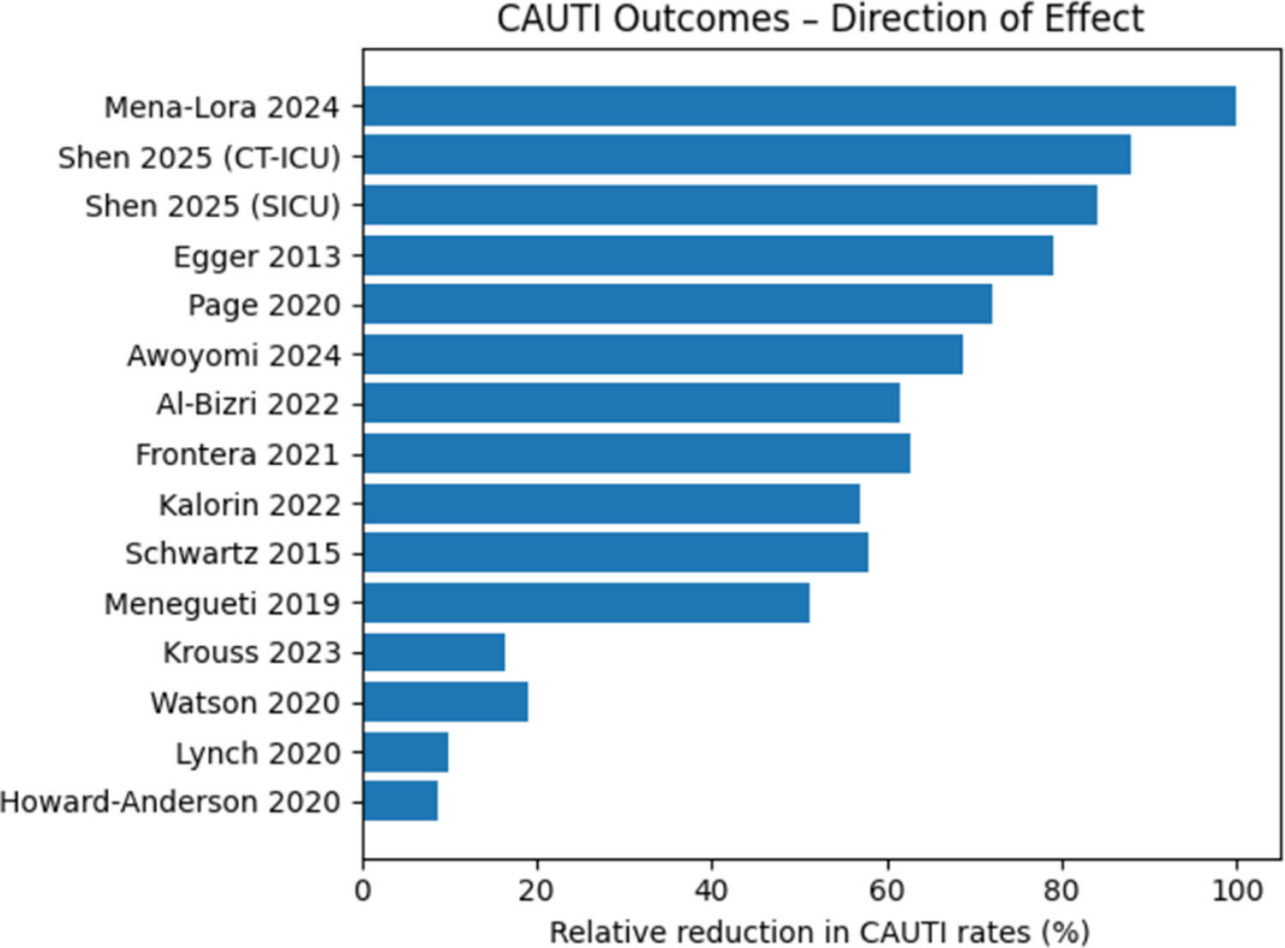

**Figure:**